# Regulation of Cardiac Cell Fate by microRNAs: Implications for Heart Regeneration

**DOI:** 10.3390/cells3040996

**Published:** 2014-10-29

**Authors:** Margarida Gama-Carvalho, Jorge Andrade, Luis Brás-Rosário

**Affiliations:** 1 Center for Biodiversity, Functional and Integrative Genomics, Faculdade de Ciências, Universidade de Lisboa, 1749-016 Lisboa, Portugal; E-Mail: jmiandrade@fc.ul.pt; 2 Centro de Cardiologia da Universidade de Lisboa, Faculdade de Medicina de Lisboa, 1649-035 Lisboa, Portugal; E-Mail: lsrosario@medicina.ulisboa.pt

**Keywords:** microRNAs, gene regulation, cell differentiation, cardiac regeneration

## Abstract

microRNAs are post-transcriptional regulators of gene expression that have been shown to be central players in the establishment of cellular programs, often acting as switches that control the choice between proliferation and differentiation during development and in adult tissues. The heart develops from two small patches of cells in the mesoderm, the heart fields, which originate the different cardiac cell types, including cardiomyocytes, vascular smooth muscle and endothelial cells. These progenitors proliferate and differentiate to establish a highly connected three-dimensional structure, involving a robust succession of gene expression programs strongly influenced by microRNAs. Although the mammalian heart has conventionally been viewed as a post-mitotic organ, cardiac cells have recently been shown to display some regenerative potential, which is nonetheless insufficient to regenerate heart lesions, in contrast with other vertebrates like the zebrafish. Both the proliferation of adult cardiac stem cells and the ability of cardiomyocytes to re-enter the cell cycle have been proposed to sustain these regenerative processes. Here we review the role of microRNAs in the control of stem cell and cardiomyocyte dependent cardiac regeneration processes, and discuss potential applications for the treatment of cardiac injury.

## 1. Introduction

The recent emergence of new sequencing technologies has significantly changed our understanding of the organization of eukaryotic genomes, providing exciting insights into the role of non-coding (nc) DNA sequences. Previously known as “junk DNA” [1], these ubiquitous entities are now acknowledged to play critical roles in the regulation of gene expression and, not surprisingly, there is increasing evidence that directly correlates their relative abundance to the complexity of higher organisms [2,3,4,5]. It is now clear that over 90% of the human genome is transcribed generating a wide variety of non-coding transcripts (around 9.000 small-ncRNAs, 10.000-32.000 long-ncRNAs and about 11.000 pseudogenes [6,7]) that vastly exceed the number of coding transcripts (21.000 coding genes). The relevance of the non-coding transcriptome in the complex regulatory networks that contribute to tissue homeostasis and organismal complexity is becoming increasingly apparent. Several studies have uncovered critical roles of the ncRNome since they act as master regulators of cell fate and function at all levels, from epigenetic control to mRNA translation and cell-to-cell communication. Non-coding transcripts can be classified into two major classes based on their relative size. Among the small non-coding RNAs, microRNAs (miRNAs or miRs), a class of 21 to 24 nucleotide (nt) long ncRNAs, stand out as one of the best characterized families, with the current count for the human genome standing at 2588 mature miRs in the latest version of miRbase (v21) [8]. It is likely that the known diversity of this family will continue to increase, as attested by the recently reported discovery of 2469 novel human miRNA candidates [9], although for a vast majority of annotated miRNAs their functional relevance remains unclear. Nevertheless, this repertoire is believed to greatly enhance the complexity of the regulatory layers that control temporal and spatial gene expression. 

Like many other organismal processes, mammalian heart development and homeostasis have been increasingly shown to be tightly regulated by miRNAs. However, unlike other mammalian organs or the heart of lower vertebrates, the mammalian heart displays very little regenerative abilities. Cardiac dysfunction resulting from myocardium cell death, as in aging or myocardial infarction, is therefore a major health problem in urgent need of new therapeutic solutions. During the past decade, several studies have come to suggest that the potential for cardiac regeneration may still be present, albeit silenced, in the mammalian heart [10,11,12]. Therefore, novel insights into the role of the tiny molecular switches that can play determinant roles in cardiac cell proliferation and differentiation are of great relevance, not only to complement the current understanding of heart biology, but also to open new windows for the development of innovative strategies to treat several cardiac-related pathologies. 

In this review, we will focus on the role of miRNAs as master regulators of cardiac development, cell fate and proliferation and discuss how recent advances in our understanding of the heart’s structure and function as well as novel discoveries in the field of cell fate reprogramming are bringing these small molecules to the forefront of regenerative therapies for heart injury. A related field with high potential for cardiac repair – cell therapy involving the transplantation and *in situ* differentiation of stem cells [13,14]—in which miRNAs play a relevant role as modulators of both pluripotency and differentiation [15], will not be discussed here in detail. 

## 2. Regulatory Programs Underlying Heart Development

Organ formation involves the sequential deployment of gene regulatory events that define cell fate by influencing proliferation and differentiation, while determining their physical arrangement into well-defined structures. The underlying regulatory programs need to coordinate the multiple dimensions of the process by defining the appropriate timing, spatial organization and feedback controls that are required to ensure the canalization of developmental processes. During the past decade, a significant progress in our understanding of evolutionary, developmental and genetic processes coordinating mammalian heart development has been achieved. More recently, microRNAs have been shown to be an integral part of these regulatory layers, thereby acting as key regulators of organ development. 

### 2.1. Transcriptional Networks in Embryonic Heart Development

The development of the mammalian heart is a relatively well-characterized paradigm of the establishment of such regulatory programs. Although often misconstrued as a simple muscular pump, the heart is in fact a complex organ in which several cell types—including cardiac and smooth muscle, endothelial and pacemaker cells—are integrated into a highly interconnected three-dimensional structure. A decade of studies has unraveled to significant detail the transcriptional networks that control heart development, with particular emphasis on the mechanisms underlying skeletal myogenesis. The current model identifies a primordial core of myogenic transcription factors—MEF2 and NK2—that became involved in the regulation of muscle-specific gene expression early during the evolution of animals (reviewed by [16]). With the appearance of the bilateria, these genes became integrated in a cardiogenic network with additional transcription factors—GATA, Tbx, and Hand—that evolved to regulate both cardiogenic differentiation, including the expression of contractile proteins, and the morphogenesis of simple cardiac structures [16]. The appearance of a multi-chambered, asymmetric heart was marked by duplications and specializations of several of these genes, in association with the appearance of complex morphogenetic patterns that lead to the formation of the organ during development. For example, the two ancestral GATA genes present in the bilateria (GATA1/2/3 and GATA4/5/6) gave rise to a total of six genes (GATA1 to 6) as a consequence of the genome duplication events that occurred during vertebrate evolution [17]. Of these, GATA4, GATA5 and GATA6 have been shown to the be expressed in the heart and to be implicated in heart development [16]. Of note, the evolutionary retention of all these paralogous genes is quite remarkable, as a comparative study between the amphioxus and the human genome suggests that only about ¼ of the human genes correspond to duplicated genes, with a much smaller fraction showing the retention of multiple paralogs [18]. Therefore, the expansion of the cardiogenic transcriptional machinery must have been supported by a strong evolutionary pressure, likely related to its critical role in the development of an increasingly complex heart. By week 8 of human development, this highly coordinated morphogenetic program will have lead to the establishment of the basic heart structure. During the period of time that follows until birth, heart development will focus on an unparalleled increase in size. In humans, this means the heart will become roughly 10000× larger than its mouse counterpart, involving a much longer developmental time frame (several weeks, compared to 48h). Recent studies suggest that this is achieved by a ‘stem cell’ based mechanism rather than by division of differentiated cell types [19,20].

### 2.2. A Stem Cell Model for Heart Development

The pluripotent stem cell paradigm for heart development has been established from multiple lines of evidence. Lineage tracing in developmental models have clearly shown that the myocardium, with all its different cell types, is formed primarily from two patches of mesoderm present in the early embryo, termed the first and second heart fields (FHF and SHF), which deploy slightly different gene expression programs during development (reviewed by [20]). Cells from the SHF will contribute to over 70% of the myocardium, whereas the FHF is the only source of cells for the left ventricle (see below). Two additional embryonic regions, the cardiac neural crest and the proepicardium have also been shown to provide smaller contributions to the heart structure. The first gives rise to the vascular smooth muscle of the aortic arch, ductus arteriosus and the great vessels and essential components of the cardiac autonomic nervous system, while the second generates the epicardium tissue that surrounds the heart and contributes to the coronary vasculature, as well as providing an additional source for cardiomyocytes [21]. 

**Figure 1:**
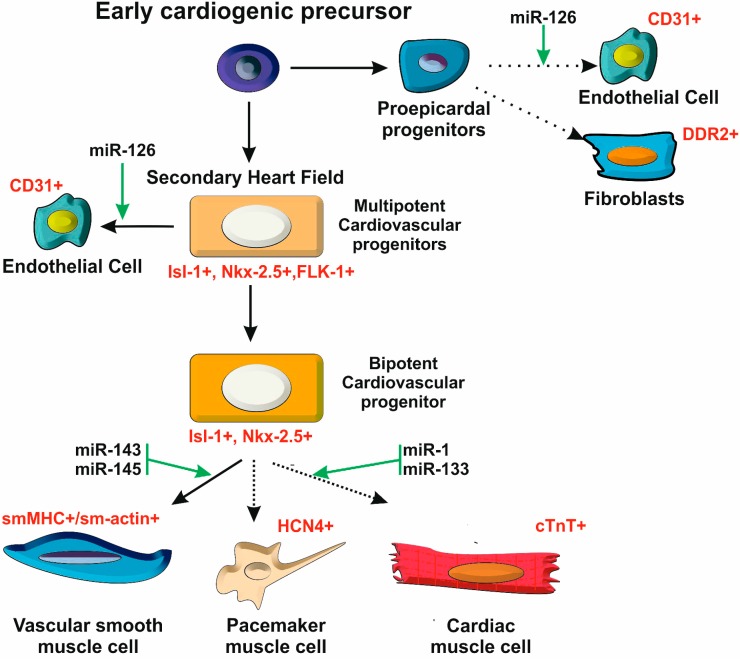
A stem cell model for heart development. The heart is composed of different cell types that are generated from multipotent cardiac progenitors. Expression of the LIM-homeodomain transcription factor Islet-1 (Isl-1+) is a hallmark of these cardiac progenitors. The diversification of heart cell lineages is acknowledged to be controlled by several miRNAs.

Although the full details of the specification of cellular lineages that compose the heart remain to be worked out, several lines of evidence support the view that the process follows the same logic as the development of the hematopoietic system, in which a multipotent progenitor gives rise to the different specialized cell types through successive steps of lineage commitment (Figure 1). The expression of different transcriptional activators and downstream target genes along this differentiation series highlights the underlying regulatory programs that contribute to cardiac cell fate decisions [20]. Interestingly, the past few years have revealed that miRNAs (and other non-coding RNAs) are highly integrated into these regulatory programs, contributing to the definition of cardiac cell fate as well as to all other dimensions of heart development and function.

### 2.3. Postnatal Heart Development

After birth a significant reorganization of mammalian heart structure will take place as an adaptation to the changes in blood circulation and functional requirements for adult heart function. This reorganization involves macroscopic alterations in the heart structure and at the same time a significant remodeling of cardiomyocyte gene expression programs, leading to a transition from fetal to adult genes and protein isoforms that affects cell structures and functions as diverse as contractile fibers and energy producing pathways. This switch occurs concomitant with the almost complete cessation of cellular proliferation [22,23]. Accordingly, the mammalian heart loses most of its regenerative capacities not long after birth, dealing with stress and damage mostly through hypertrophy of pre-existing cardiomyocytes, fibroblast accumulation and scarring. Interestingly, while these responses seem to involve the re-enactment of parts of the fetal heart program they often result severe contractile dysfunction, to the point of heart failure. The regulatory mechanisms underlying the fetal to adult switch and conversely the switch to fetal expression profiles upon injury, are only beginning to be understood, but are of extreme importance for understanding the mechanisms that control heart regeneration and response to injury. As with other aspects of heart development, these switches have been recently shown to be under the influence of miRNAs. Understanding the mechanisms of biogenesis and mode of action of these molecules is therefore critical to an in-depth knowledge of many of the molecular events underlying cardiac function and repair.

## 3. microRNAs: from Biogenesis to Post-Transcriptional Control of Gene Expression

miRNA biogenesis is acknowledged to be regulated either at transcriptional [24] or post-transcriptional level [25]. During the past few years, significant progress was made regarding the systematic identification of miRNA genes, understanding of their organization and of the biogenesis mechanisms required for their synthesis and basic modes of action. It is, however, clear that many of these processes are only understood in a relatively superficial manner, and the diversity of mechanisms that have increasingly been discovered suggests that there is still much to be learnt about these small molecules.

### 3.1. microRNA Gene Structure

**Figure 2:**
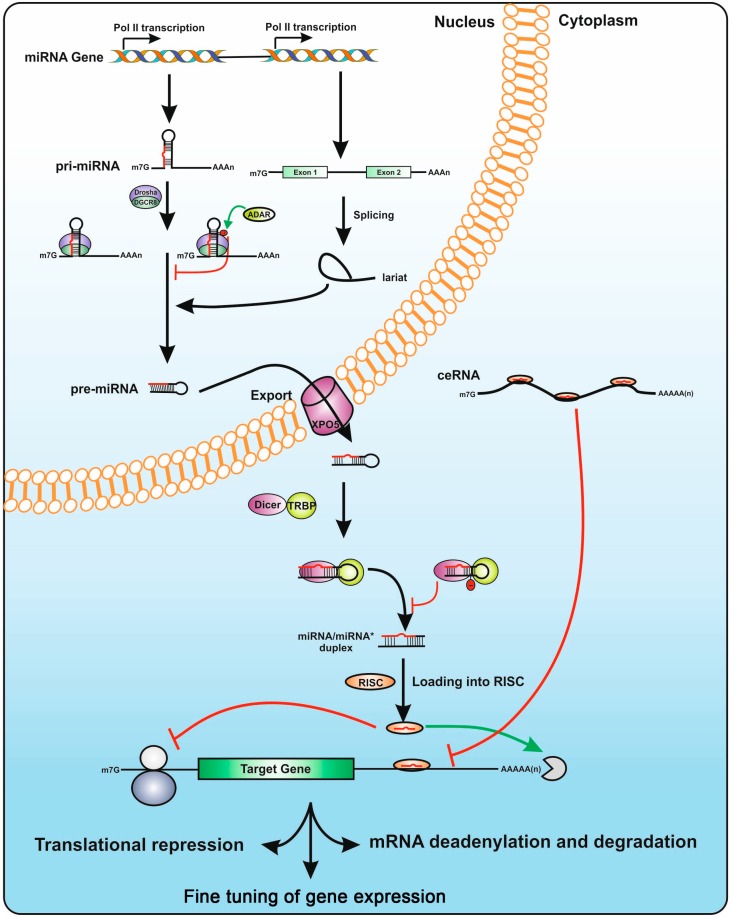
Overview of the miRNA biogenesis pathway. miRNA genes are transcribed in the nucleus by RNA Pol II as long pri-miRNA transcripts that are 5′ capped and 3′ polyadenylated. The pri-miRNA sequence folds into a hairpin loops structure that is recognized and processed by the Microprocessor complex Drosha-DGCR8, generating a pre-microRNA. *Mirtrons*, a class of unconventional miRNAs, are encoded in small introns and do not require Drosha processing. The intron lariat excised by the spliceosome is refolded into a pre-miRNA hairpin loop. The pre-miRNA is exported from the nucleus to the cytoplasm by exportin 5 (XPO5), where is further cropped by Dicer in complex with TRBP, yielding a ~22 nt double-stranded RNA called miRNA/miRNA* duplex. The functional mature miRNA is loaded together with Argonaute proteins into the RISC complex, guiding RISC to silence a target mRNA through translational repression or deadenylation. The biogenesis pathway of miRNAs are post-transcriptionally controlled by RNA editing. A-to-I editing of the miRNA’s precursors, a reaction catalyzed by ADAR enzymes, can block Drosha and Dicer processing, and thereby regulates the availability of mature miRNA in the cell. Competing endogenous RNAs (ceRNAs) can regulate mRNA expression levels by competing for shared miRNA binding sites.

With very few exceptions [26,27], miRNA genes are initially transcribed in the nucleus by the RNA polymerase II as long 5′-capped and Poly-A tailed pri-miRNA precursors, which fold into hairpin structures that are recognized by the miRNA biogenesis machinery (Figure 1) [28,29]. Based on their genomic localization, pri-miRNAs can be transcribed either from intergenic or intragenic genomic environments. The majority of mammalian miRNA genes are located either in introns of protein-coding genes or as independent non-coding transcriptional units (TUs) [30]. Within both groups, some miRs are organized into clusters transcribed as polycistronic TUs (ranging from 2 to 19 miRNA hairpins in tandem) that, upon processing by the miRNA biogenesis machinery generate multiple mature miRNAs (Figure 2A). Although initially believed to be co-transcribed and co-expressed with their host genes under the transcriptional control of the host gene promoter [30], several studies indicate that some intronic miRNA genes may not follow this rule, relying on an additional layer of transcriptional control by their own independent promoters [31,32]. A subset of intronic miRNA genes that are transcribed in an anti-sense orientation with respect to their host gene were also found to have specific *cis*-regulatory elements and thus not to depend directly on host gene expression [33,34]. Despite the complexity involving the identification of miRNA genes promoters and putative transcription factors binding sites [35], independent intronic promoters are acknowledged to be an important functional feature that allows miRNA expression levels to be controlled in a tissue- or development-specific fashion [36]. Furthermore, intronic miRs do not seem to depend on splicing of their host intron for removal [37].

### 3.2. The microRNA Biogenesis Machinery

**Figure 3:**
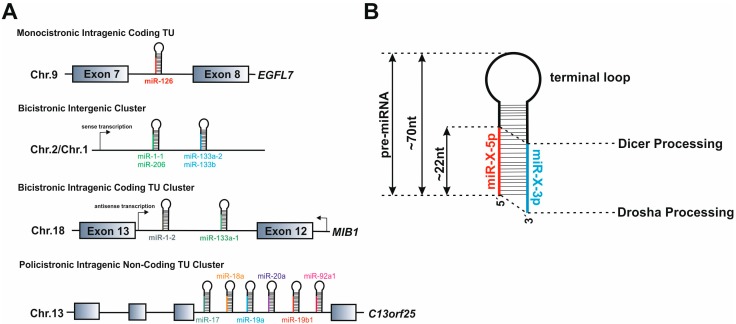
miRNA genomic organization and pre-miRNA hairpin loop features. (**A**) Mammalian miRNA genes are encoded in defined transcription units (TUs) that based on their genomic localization can be classified into three major groups: (i) intronic miRNA genes in protein-coding genes; (ii) intergenic miRNA genes; (iii) intronic miRNA genes in non-coding TUs. miRNA genes can be clustered in a single polycistronic transcript that can be processed in order to generate two or more mature miRNAs. Some miRNA genes are located within TUs with the same transcription orientation as the host gene, whereas others can be transcribed in the anti-sense orientation. (**B**) miRNA precursors fold into a hairpin loop structure that is sequentially processed. Drosha cleaves the pri-miRNA at the base of the stem-loop, generating a pre-miRNA (~70 nt long) precursor that is further processed by Dicer in order to produce a miRNA double-stranded duplex (~22 nt). Both Drosha and Dicer processing generates a characteristic 2 nt 3'overhang. Depending on the relative localization in the hairpin-loop, the mature miRNA can be termed -5p or -3p.

Upon transcription, pri-miRNAs are submitted to two sequential processing events that trim the transcript in order to yield a mature miRNA. Within the nucleus, pri-miRNAs are cropped into a 60–100nt hairpin-structured precursor (the pre-miRNA) by a multiprotein Microprocessor complex that includes the RNAase III Drosha and the DGCR8 (DiGeorge syndrome Critical Region 8) protein, as well as several auxiliary cofactors that coordinate activity and specificity of Drosha cleavage (for review see [25]). Binding of DGCR8 to the pri-miRNA allows precise positioning and orientation of Drosha’s catalytic center ~11nt upstream of the stem-loop, in order to generate a double-stranded pre-miRNA with a 2 nt 3' overhang [38]. After nuclear processing, the pre-miRNA is exported to the cytoplasm by Exportin-5 (XPO5) via a RAN-GTP dependent mechanism [39,40]. Efficient recognition by XPO5, defined not only by the length of the double stranded stem but also by the 3'overhangs, protects pre-miRs from degradation, allowing exporting of only correctly processed miRNA precursors [41,42]. In the cytoplasm, the pre-miRNA is further cleaved near the terminal loop by Dicer, another RNAse III enzyme that, together with TAR RNA binding protein (TRBP), protein kinase RNA activator (PACT) and Argonaute (Ago) proteins, forms the RNA induced silencing complex (RISC) [43,44,45]. Dicer is considered to act as a molecular ruler, cleaving the pre-miRNA at a specific distance from the ends produced by Drosha and generating a ~22nt double-stranded miRNA duplex (miR/miR*) with a 2 nt 3' overhang (Figure 2). The RISC complex incorporates one of the strands of the diced miRNA duplex, generally the one with the lowest base-pairing stability at the 5' end, while the other strand, originally termed miR*, is degraded. There are however many instances where both strands can be found as part of miR-RISC complexes, albeit at different frequencies (which may be as high as 100:1) [46]. In this case, the two miRNA strands arising from the same precursor are termed -5p or -3p, depending on their relative position in the hairpin sequence (Figure 3B). 

Although the vast majority of miRs follow the canonical biogenesis pathway, a small subset has been shown to bypass some steps. *Mirtrons*, a group of unconventional miRs, are processed by the spliceosome and do not rely on Drosha to generate pre-miRNA precursors [47]. There are also mirtron-like splicing-independent miRNAs, termed *simtrons*, which are processed by Drosha and do not require DGCR8 nor Dicer for their biogenesis [48]. Additionally, processing of some small nucleolar RNAs (snoRNAs), transfer RNAs (tRNAs) and endogenous short hairpin RNAs (shRNAs) is also reported to generate miRNA-like molecules that are independent of the Microprocessor complex [49,50,51]. 

Regulation of miRNA biogenesis is also reported to be post-transcriptionally controlled through RNA editing of miRNA transcripts by Adenosine Deaminases that act on RNA (ADARs). These enzymes catalyze the conversion of adenosine (A) into inosine (I). A-to-I editing of some miRNA precursors not only controls several steps of the biogenesis pathway, from blocking Drosha-DGCR8 cleavage to inhibition of the Dicer-TRBP complex, but may also redirect mRNA target recognition [52,53,54,55,56]. However, a recent report shows that ADAR1 and Dicer directly interact in protein-protein complexes that, independently of the deaminase activity, promote pre-miRNA cleavage by Dicer and facilitate miRNA loading into the RISC [57]. Although many questions remain to be answered, RNA editing is currently acknowledged as an effective post-transcriptional mechanism that regulates miRNA biogenesis and activity.

### 3.3. microRNA Modes of Action

The mature miRNA incorporated into RISC interacts with the mRNA through Watson-Crick complementary base-pairing that most frequently occurs at the 3' UTR, although some can bind the 5' UTR [58]. The mRNA-miRNA base-pairing interaction is primarily determined by the so called ‘seed-sequence’, a 7 nt stretch located at positions 2 to 8 of the mature miRNA. Target cleavage is generally associated with full complementarity with the full miRNA sequence, a rarely observed event in animals, where miRNA target sites are generally classified into two categories: 5' dominant, which base-pair precisely with the seed with or without additional involvement of 3' nucleotides; and 3' compensatory sites, in which the base pairing of 3' miRNA nucleotides is required to compensate for insufficient seed complementarity [46]. miRs that share the same seed sequence are often defined as a ‘family’. In many cases, these miRNAs arise from paralogous genes. Examples include the let-7 family, which contains the first miRNA to ever be described [59] or the miR-1/133 and miR-15 families. 

Binding of the RISC complex to a mRNA generally results in a down regulation of target gene expression, either through ‘dicing’ of the mRNA (endonucleolytic cleavage), which is relatively rare in animals, or translation inhibition and degradation through decapping and deadenylation [60]. Translation inhibition may occur in a reversible fashion, often associated to the accumulation of the mRNA-miRNA-RISC complex in cellular structures termed P-bodies [46]. Quantitative proteomic analysis suggests that although in some cases target mRNA translation can be repressed without detectable changes in mRNA levels, most mRNAs displaying strong (*i.e.*, over 30%) reduction in protein levels also display detectable mRNA destabilization [61,62]. The mechanistic details and relative contribution of each process to gene silencing by miRs has been the object of intensive investigation, but several critical aspects remain to be resolved (reviewed by [60,63]). In spite of the undisputed relevance of the seed sequence in determining the miR-mRNA base pairing interaction, a recent report suggests that imperfect centered binding sites may be both common and functional in human cells [64]. The ability of a miRNA to interact with a target sequence is further influenced by the secondary structure of the target and the association of RNA binding proteins (RBPs) [65,66,67]. Once these interactions are established, the diverse nature of the outcomes on gene expression programs determines a whole new level of regulation that can have quite a profound impact on cell function and fate.

## 4. microRNAs’ Biological Functions: Getting to the Heart of the Matter

A basic knowledge of how miRNAs interact with their target mRNAs and the consequences of this interaction in terms of target gene expression is still one step away from understanding the biological impact of these regulators on cell fate and function. Again, the diversity of modes of action, complex underpinnings of the system and in many cases, the technical difficulties associated with the required experimental studies impose significant limitations on the quest for understanding the function of miRs at the level of the organism.

### 4.1. Regulation of Gene Expression Programs by microRNAs

The presence of multiple target sites with different affinities and abundances within a given cell (or even on a single mRNA) creates a dynamic environment that will strongly influence the interaction kinetics between miRNA and mRNA. To make matters more complex, recent data has revealed the existence of several cellular RNA molecules that act as miRNA sponges to regulate their availability. These include pseudogene mRNAs [68], long-non-coding RNAs [69] and even previously unknown circular RNA species [70,71]. Together with the properties of the miR-mRNA interaction, this makes the sequence-based prediction of effective miRNA targets a complex problem that many groups have attempted to tackle with limited success. In particular, the integration of kinetic models into predictions will likely be fundamental for a robust prediction of functionally relevant mir-mRNA interaction (see discussion by [72]). As a corollary of all this, the outcome of a miR-mRNA interaction can be markedly different, ranging from full repression of gene expression through the reduction of target mRNA abundance to inconsequential levels (termed ‘switch targets’), to reduction of target abundance (or translation rates) to lower, yet functional levels (termed ‘tuning targets’), to apparently having no effect (termed neutral targets) [73].

Tremendous efforts have been made in recent years in order understand miRNA biology, with functional studies pointing to important roles played by miRs in the regulation of almost every cellular process. Additionally, the misregulation of miRNAs is often associated with human pathologies [74]. 

In theory, a single miRNA can interact with hundreds of mRNA molecules and a specific mRNA molecule may be the target of multiple miRs. Therefore, miR-mRNA interactions can define complex regulatory networks that serve to coordinate entire gene expression programs. These networks may further involve intercellular interactions according, for example, to the reports that miRs are actively secreted in exosomes or are capable of intercellular movement through a gap junction dependent mechanism, like the ones present in heart cardiomyocytes [75]. The current knowledge of miRNA biology has further revealed complex regulatory events in which transcriptional factors and miRNAs interplay through positive and negative feedback loops in order to control gene expression programs that modulate cell fate and differentiation (Figure 3).

### 4.2. Role of microRNAs in Cell Fate Decisions

The observation that the miRNA biogenesis machinery, although required for vertebrate development, was not essential for cell survival and did not interfere with the early stages of body plan establishment led to the suggestion that the main role of miRs might be related to regulating the diversification of cell types within organs and tissues [76,77]. The first studies identifying miRNAs as regulators of lineage commitment in animals came from studies involving over-expression of tissue enriched and tissue specific miRs. The latter can be defined as miRs whose expression level in one tissue is 20 fold or more higher than in all other tissues [78]. The hematopoietic enriched miR-181 was the first to be shown to shift progenitor cell differentiation into the specific lineage where it is abundant—B cells [79]. However, this study was specifically performed in the context of hematopoietic progenitors and did not provide significant insights into the underlying regulatory mechanisms. A more defined view of the power of miRs to shift gene expression programs into cell type specific programs came from the transfection of miR-124 and miR-1 into HeLa cells, followed by microarray analysis [80]. These miRs display a highly tissue specific expression pattern, with miR-124 being preferentially expressed in the brain and miR-1 in the skeletal muscle and heart. The analysis of the impact of overexpressing these miRs in HeLa cells established for the first time that animal miRs can influence the abundance of over 100 target mRNAs through base-pairing interactions between the seed and the 3' UTR, as opposed to having only an effect on translation repression [80]. Furthermore, Lim and colleagues found that they could infer the tissue of origin of these miRs by simply comparing the gene expression profile of the transfected cells with the profiles of different human tissues. Indeed, the gene expression profile was significantly and specifically shifted towards the expression profile of the miRNA tissue of origin through the systematic silencing of genes that were not expressed in those tissues. This led to the proposal that miRs help define and maintain the different cell types of animals. This study was complemented by the analysis of the impact of inactivating miR-1 expression in Drosophila, which revealed the extent of conservation of miRNA functions [81]. miR-1 knock-out (KO) led to major alterations in myofiber structure and muscle growth, resulting in larval paralysis and lethality. Interestingly, this phenotype could be rescued by muscle specific expression of miR-1, reinforcing its tissue-specific nature. Later experiments involving the ectopic expression of miR-1 in mouse and human embryonic stem cells revealed that this miRNA is a strong promoter of mesoderm differentiation, displaying enhanced cardiomyocyte formation, while at the same time suppressing gene expression of other lineages [82]. Based on these results, the authors proposed for the first time that miRs could be used as tools to drive the differentiation of embryonic stem cells. Further studies have identified multiple miRs that act as regulators of cell fate, as reviewed by Ivey and Srivastava (2010) [83]. In addition to this, miRs have also been shown to play critical roles in the establishment of stem cell properties, in particular in the maintenance of the pluripotent state (reviewed by [84]). 

### 4.3. miRNAs as Critical Regulators of Stem Cell Properties

**Figure 4:**
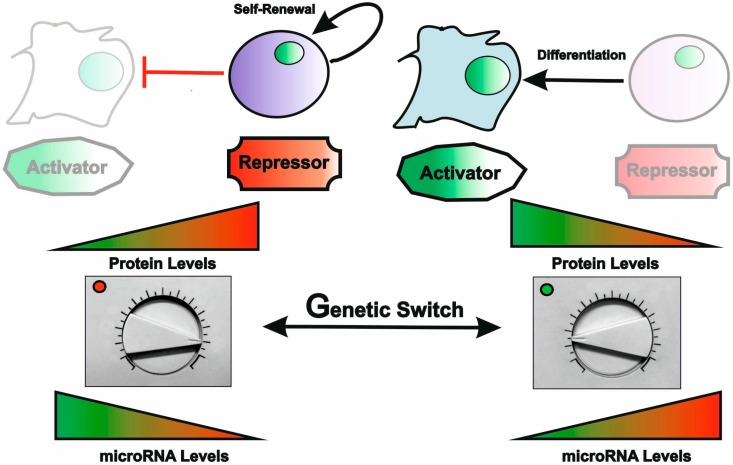
miRNAs are master regulators of cell lineage commitment. In response to a given genetic switch, miRNAs can fine-tune the transcriptome to modulate cell proliferation and differentiation. By targeting positive or negative regulators of lineage specification, miRNAs act as rheostats that adjust the proteome required for the activation or repression of the genetic programs that provide robustness to cell fate decisions.

Embryonic stem cells (ESCs) and tissue or adult stem cells are central mediators of tissue development and homeostasis, respectively. By definition stem cells have the property of asymmetric division, *i.e.*, they divide and generate another cell with stem cell properties by self-renewal, and a cell committed to specialized functions through differentiation. The diversity of cell types that can be generated from such a self-renewing cell may range from unlimited (for the totipotent ESCs) to different degrees of pre-commitment in adult stem cells, which may thus be referred more appropriately as progenitor cells. ESCs were studied in depth for the role of miRs in the definition of a cellular identity and control of their characteristic properties. Inhibition of every miRNA present in the cell, by suppression of Dicer, led to an acute loss of proliferative potential and a failure to silence the embryonic cell program and differentiate [85]. ESCs have a defined miRNA profile with a limited number of expressed miRs, both in humans and in mice, that decrease as stem cells differentiate [85]. Interestingly, some of the identified miRs present in ESCs are involved in the negative regulation of cell proliferation and of the pluripotency factors Oct4, Nanog and Sox2. Overall, ESCs seem to express miRs with antagonistic functions in the regulation of self-renewal and proliferation [84]. Depending on their balance, an ensuing cell fate decision will be made (Figure 4). In spite of being often viewed as ‘fine-tuners’ of gene expression or ‘robustness’ enforcers, recent studies have shown that a small number of stem cell specific miRs are ‘powerful’ enough to promote somatic cell reprogramming, reverting cells back to an ESC like state [86,87,88]. Conversely, some tissue-specific miRs have been shown to be able to direct trans-differentiation (i.e., the direct reprogramming of cell state without passing through an undifferentiated condition) [89,90]. These observations have placed miRs under the spotlight as emerging tools for the development of regenerative therapies (reviewed in [15,91]).

### 4.4. Regulatory Feedback between miRNAs and Transcription Factors

The properties that allow miRNAs to have such a robust impact on the definition of cellular gene expression programs, seem to stem from the interweaving of their post-transcriptional regulatory activities with core transcriptional networks that control cell fate and differentiation [83]. Although the specific modalities by which the two intersect can be quite diverse depending on the specific situation, a common theme of feedback and feed-forward loops between miRs and transcription factors, either counterbalancing or reinforcing cellular decisions, has clearly emerged.

**Figure 5:**
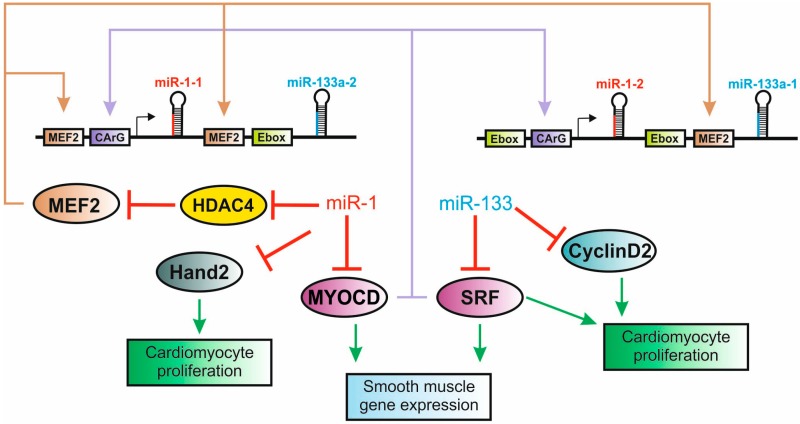
Transcriptional regulatory networks controlled by miR-1 and miR-133 during cardiac muscle differentiation. In mammals, the duplicated miR-1/miR-133 locus is transcribed into a bicistronic transcript that is regulated by multiple independent upstream intronic enhancers. In the embryonic heart, expression of the miR-1/miR-133 locus is transcriptionally regulated by the myogenic transcription factors SFR, MYOCD and MEF2. By targeting the same transcription factors that regulate miR-1/miR133 expression and control cardiac progenitor cell proliferation and differentiation, miR-1 and miR-133 fine-tune multiple nodes of the genetic networks that control cardiac muscle differentiation.

One of the first known examples of these regulatory loops involves the interplay of miR-1 and the cardiogenic and myogenic transcription factors (Figure 5). miR-1 is actually a member of an evolutionarily conserved family that in mammals is organized as three bicistronic TUs (Figure 3A). miR-1 is encoded by two genes, miR-1-1 and 1-2, which are clustered with miR-133a-2 and miR-133a-1, respectively. These genes are under the control of the master cardiogenic and myogenic transcription regulators SRF, Mef2, MyoD and myogenin, thereby presenting both cardiac and skeletal muscle specific expression [34,92,93]. A third paralogous gene cluster encodes the miR-206/miR-133b pair, which is only expressed in the skeletal muscle. miR-1 and miR-133 are for the most part co-expressed and contribute to the establishment of a muscle specific gene expression program while having somewhat antagonistic roles in the control of proliferation and differentiation [94]. Indeed, while miR-1 is acknowledged to trigger differentiation of both mouse and human embryonic stem (ES) cells into the cardiomyocytes, miR-133 was found to act in partial opposition to miR-1, by promoting muscle progenitor expansion and preventing terminal differentiation [82]. Interestingly, both miR-1 and miR-133 have been shown to be negative regulators of the same cardiogenic transcription factors that, in addition to promoting their expression, activate protein-coding genes involved in muscle function (e.g., sarcomere genes) [34,92,94,95]. The existence of multiple independent enhancers of the miR-1/133 genes and the negative feedback loop established between miRs and transcription factors allow fine-tuning of temporal-spatial control of gene expression, providing a means of reinforcing the cardiac and skeletal-muscle-specific programs during development and cell differentiation.

## 5. microRNAs in Heart Development

The earliest studies on miRNA expression and function immediately suggested a prominent role in the cardiac tissue [96,97,98,99]. This initial hypothesis was to be supported by a growing number of studies that showed not only that the miRNA biogenesis machinery is required for normal heart development, but were also able to dissect the roles of specific miRNAs in the regulatory networks controlling the embryonic and post-natal steps of heart development.

### 5.1. Requirement of the microRNA Machinery for Heart Development

The first evidence for the role of miRs in heart development came from the study of knock-out animals targeting the core miRNA biogenesis machinery: Drosha, DGCR8, Dicer and Ago2 [100,101,102,103,104]. The use of mouse models is limited by the fact that the ablation of these essential proteins results in early embryonic lethality, preventing the analysis of the overall contribution of miRs to the cardiovascular system. Therefore, addressing the specific contributions of the miRNA pathway to heart development required the use of tissue/cell type specific knock-outs for these genes. Deletion of Dicer during early heart development has been achieved using Nkx2.5 driven recombinase expression [100,101]. These studies reported major defects in ventricular myocardium structure, cardiac outflow tract morphogenesis and chamber septation, in addition to pericardial edema. Deletion of Dicer at later stages in development using the cardiomyocyte specific promotor for the MHC/Myh6 gene resulted in early post-natal death associated to the presence of cytoskeletal defects and deregulation of proteins important for contractility, cardiac conduction, and calcium handling [102]. Dicer and Dgcr8 deletion in neural crest cells, which participate in the development of the cardiac outflow tract, revealed critical contributions of miRs for cell migration and patterning processes [103,104]. Interestingly, the observed defects strongly resembled the developmental abnormalities present in some human genetic disorders, including the Di George micro-deletion syndrome, which includes the genomic locus of the DGCR8 gene that is part of the canonical miRNA processing machinery [105]. Although the heart malformations associated with this syndrome have been proposed to result from haplo-insufficiency of the cardiogenic transcription factor Tbx1, also located in the Di George critical region [106,107], it is highly likely that part of these defects are in fact linked to perturbations in the miRNA biogenesis pathway. 

While suggesting an important role for miRs at all levels of regulation of the cardiac developmental program, from differentiation to morphogenesis, these approaches fail to identify the contributions of specific miRs, which require their targeted manipulation. Together with studies characterizing miRNA expression levels across tissues and developmental stages and in response to heart injury, the development of these functional approaches led to the identification of multiple miRs that play a role in heart development and response to injury, a topic that has been the subject of several recent reviews [94,108].

### 5.2. The miR-1/133 Family is a Critical Component of Cardiogenic Regulatory Networks

Standing out among the miRs with roles in cardiac function is the highly conserved miR-1, originally identified in early studies in *Drosophila* and *C. elegans* and shown to be highly expressed in the human and mouse heart and skeletal muscle [96,97,98,99]. As discussed above, miR-1 was later found to be encoded in two bicistronic clusters together with miR-133a, which displays the same overall expression pattern and is also a key regulator of muscle and cardiac cell differentiation. 

Zhao and colleagues where the first to demonstrate that miR-1 can influence heart development, by showing that miR-1 overexpression in the developing mouse heart down-regulates the cardiogenic regulator Hand2 and results in premature withdrawal of cardiomyocytes from the cell cycle [34]. Strikingly, deletion of the single miR-1 gene in Drosophila also resulted in a spectrum of defects in muscle and cardiac differentiation [81,109]. These studies revealed for the first time a specific role for tissue-specific miRs in the establishment of cell differentiation programs, showing that miR-1 is required to maintain muscle gene expression and suggesting a high degree of functional conservation between flies and mammals. 

Interestingly, miR-133 is also conserved in Drosophila, but it is not clustered with the single dm-miR-1 gene. Studies on the evolution of this miRNA family suggest that they became clustered in the early stages of chordate evolution, becoming linked to the GATA4/5/6 ancestral gene before the genome duplication events that occurred near the base of the vertebrate lineage [110]. The genomic duplication events at the base of vertebrate evolution therefore resulted not only in the appearance of the three cardiogenic GATA genes (GATA4, GATA5 and GATA6) but also created the miR-1/133 gene family. One of the miR-1 paralogs was later converted into the skeletal muscle specific miR-206, roughly at the time of the teleost divergence [110]. Strikingly, the association between GATA-4 and the miR-206/133b gene cluster was lost during mammalian evolution, although it is still retained in all other vertebrates. It is, therefore, tempting to speculate that the evolution of the gene regulatory programs underlying the development of the mammalian heart, which involved the expansion and re-organization of the core cardiogenic transcriptional machinery [16], occurred in tight connection with the development of a miRNA dependent control layer. 

Further insights into the role of the miR-1/133 family in cardiac development were obtained through the generation of knock-out mice with targeted ablation of these miRs. miR-1 was one of the first miRs whose function was characterized by this approach [101,111]. In spite of its duplication, ablation of the 21 nt miR-1 sequence in the miR-1-2 gene using a targeted recombination strategy designed to replace it with a Neo-LacZ selection marker was reported to resulted in 50% embryonic lethality with ventricular septation defects (VSD) [101]. The surviving animals had normal heart morphologies but displayed heart hyperplasia associated to abnormal myocyte proliferation and cardiac conduction defects, with frequent sudden death during the first post-natal weeks. Similar results have been reported for a miR-1-1 KO using the same targeting strategy [112]. However, the apparent haplo-insufficiency of miR-1 in heart development has been questioned by more recent double KO mice, which do not show any embryonic lethality [113]. The key difference between this and the earlier studies is the removal of the positive selection cassettes. These sequence elements have been reported to cause transcriptional interference and could thereby affect the KO phenotype in a non-specific manner [114]. An older study using knock-outs for miR-133a-1 and miR-133-a2 with removal of the selection markers also supports the redundancy of these genes during heart development, as only the double knock-out mice displayed detectable cardiac phenotypes [115]. Finally, a more recent targeted ablation of a single miR-1/133 cluster did not show any significant developmental or cardiac defects, which were only observed upon deletion of both clusters [116]. 

These more recent models also raise interesting questions regarding the role of the miRs-1/133 during heart development. Indeed, Wei and colleagues do not report any embryonic lethality in the mir-1 dKO mice [113]. For the miR-133 dKO animals, albeit a modest reduction in viability is observed during embryonic development, along with a high number of VSD related deaths soon after birth (day P0/P1), about half of the mice hearts developed with a relatively normal morphology [115]. Only the miR-1/133 dKO displayed significant cardiac abnormalities during embryonic development, with no animal surviving past embryonic day E10.5 [116]. This is in sharp contrast with the previously described defect of miR-133a dKO mice, which becomes apparent only at later stages [115]. The complete loss of miR-1/133a did not interfere with formation of the primary heart tube, but affected maturation and further specification of embryonic cardiomyocytes during expansion of the compact layer of the myocardium.

### 5.3. microRNAs Play a Critical Role in the Cardiac Fetal-to-Adult Switch

In spite of the fact that the available KO mice for the mR-1/133 family present some discrepancies in the observed phenotypes, the requirement for these miRs for an appropriate transition from the fetal to a more mature/adult cardiac gene expression program is extremely consistent. Interestingly, the lack of miR-1/miR133 seems to affect multiple cellular pathways required for this transition. These include marked changes in the cardiac contractibility apparatus, with a switch from fetal specific to adult isoforms of several sarcomeric proteins, and the silencing of smooth muscle proteins expressed early during cardiomyocyte differentiation, which appears to be regulated by both miR-1 and miR-133. In addition, this switch is associated to significant changes in energy metabolism, moving from glycolytic pathways in fetal cardiomyocytes to fatty acid oxidation in the adult heart, which seems to be also affected in the absence of miR-1. Finally, the cessation of cell proliferation, a hallmark of the fetal to adult transition, was also reported to be affected in four of these mouse models, where cardiomyocytes division was observed to occur late after birth [101,113,115]. Thus, the available data support the view that miR-1 and miR-133 play a critical synergistic role in the suppression the cardiac fetal gene program and enforcement of adult skeletal muscle properties, driving cardiac maturation. 

Interestingly, several other miRs are reported to play critical roles in the fetal to post-natal cardiac switch. These include the so called ‘myomiRs’, mir-208a, miR-208b and miR-499, which are encoded as introns of the α, β and 7b myosin heavy chain (MHC) encoding genes Myh6, Myh7 and Myh7b [94]. The β-MHC is expressed in the fetal heart, switching to the α-MHC in the adult heart. This switch involves a regulatory circuitry among the MyomiRs and their host myosins that appears to be operative specifically in the adult heart [117]. Additionally, cardiac postnatal development is marked by alternative splicing transitions from embryonic to adult cardiac protein proteins, coordinated for the most part by the CUBGP and ETR-3-like factor (CELF) family of splicing regulators. These proteins have in turn been shown to be directly regulated by miRs-23a/b, which coordinate a whole alternative splicing network during post-natal development [118]. Finally, the upregulation of the miR-15 family has been suggested to be a key regulatory event linked to the terminal exit of cardiomyocytes from cell cycle during the post-natal period [119]. This critical role of miRs in the establishment of a robust post-natal cardiac gene expression program is further supported by studies where the targeted post-natal deletion of Dicer was performed in the mouse heart, leading to strong re-expression of fetal genes along with a marked hypertrophic response [120]. Conversely, the cardiac response to stress is marked by the aberrant expression of multiple miRs, in many cases associated with a re-enactment of the fetal gene expression program [121,122].

## 6. Heart Regeneration: A microRNA Connection to the Lost Link

The heart is one of the mammalian organs with less regenerative potential [13,123,124,125]. As a consequence, heart function is significantly impaired by cardiac injury and aging, representing one of the most significant public health problems. This characteristic of the mammalian heart is in stark contrast, not only with the regenerative potential of many other tissues such as liver, gut, muscle or bone, but also with the cardiac regeneration abilities of other vertebrates, like amphibians or fish. Zebrafish, for example, can easily regenerate large surgical amputations of the cardiac apex, corresponding to about 20% of the total ventricular mass. This regeneration has been recently shown to occur mainly through cell division of mature cardiomyocytes [126,127], which in the adult mammalian heart display only residual proliferative activity (reviewed in [13]). Understanding the origin of these differences and how they connect to the conserved underlying genetic circuitry established by miRs and transcription factors can provide important insights into the development of regenerative therapies for human heart disease.

### 6.1. Adult Cardiac Progenitor Cells under the Control of microRNAs Provide a Limited Source of Renewal

**Figure 6:**
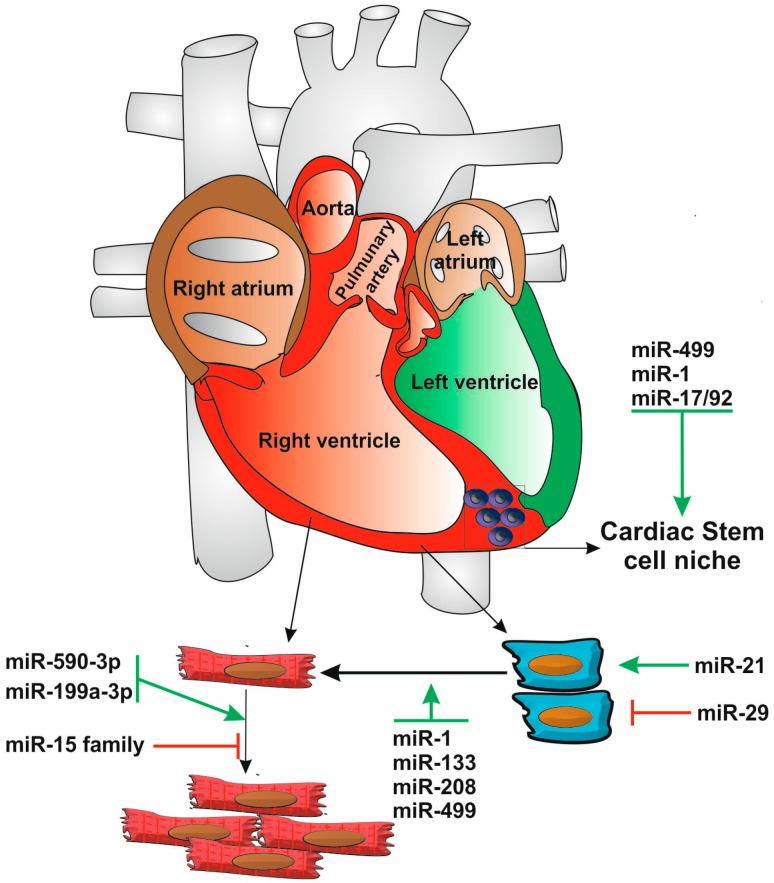
miRNAs in the definition of cardiac cell fate. Niches of cardiac stem cells in the postnatal heart have the potential to differentiate into several cellular lineages that compose the heart. miRNAs can impact on heart regeneration by modulating the cellular fate of these resident cardiac progenitor cells and other cardiac cell types, in particular cardiac muscle cells and cardiac fibroblasts. Heart regions are colored according to their developmental origin: red—first heart field; green—second heart field; brown—both heart fields. See text for details.

The adult mammalian heart has been traditionally considered to be a post-mitotic organ because in classical histology studies, cardiomyocytes were never seen to divide (although they do undergo DNA replication). Two clinical observations first raised the possibility that there is cardiomyocyte renewal during human adult life. First came the observation that after myocardial infarction there are dividing cardiomyocytes [128]; second biopsies from human heart transplants with donor recipient sex mismatch were shown to harbor newly formed cardiomyocytes from the recipient [129]. A later study used the rise of Carbon 14 levels in the atmosphere due to the test atomic explosions that took place during the cold war, to date the DNA from human biopsies and calculate the renewal rate of cardiomyocytes during the human lifespan [124]. This study confirmed the existence of cardiomyocyte renewal, suggesting an age-dependent rate ranging from 1% per year at 20 to 0.4% at 75. These observations did not address whether this renewal involves the cell cycle re-entry of cardiomyocytes or rather results from the proliferation of progenitor cells. Supporting the second hypothesis, cardiac resident cells identified by the stem cell membrane markers c-kit, Sca-1, MDR1, were isolated from both human and mouse adult hearts. These cells are clonogenic, self perpetuating, can differentiate into all cardiac cell lineages and regenerate myocardium [123,130,131,132,133] identifying them as true adult cardiac progenitors (CPs). Resident cardiac progenitor cells have been shown to participate in the maintenance of normal heart homeostasis following a clonal differentiation pathway [134]. Interestingly, genes that control cardiac development are active in CPs and their differentiation process seems to replicate the embryonic program (reviewed by [21]). However, unlike embryological cells developing into cardiomyocytes, for which once the process begins, it inexorably leads to the final phenotype, these adult progenitors manage to become stuck in an intermediate stage; both the mechanisms that stop and restart them are unknown, as well as the pathways that regulate their differentiation into the different cardiac cell types. As expected, miRNA profiling studies suggest that the CP phenotype is strongly influenced by these regulatory molecules. Indeed, human and mouse adult CPs express a subset of miRs that is clearly distinctive from cardiac embryonic, neonatal and mesenchymal progenitor cells, as well as from mature cardiomyocytes. The differentially expressed miRs are highly linked to the regulation of proliferation and differentiation processes [135,136,137]. Furthermore, the manipulation of some of these miRs *in vitro* (miR-1 and miR-499) and *in vivo* (the miR-17/92 cluster), was shown to be able to modulate CP cell fate [135,136] (Figure 6). Studies in rats have suggested that CP cells can be locally induced to proliferate and differentiate, contributing to a reversal of age and injury phenotypes [138]. Interestingly, the ability of CP-derived cardiomyocytes to fully mature and integrate into the functioning heart *in vivo* was shown to be modulated by miR-499, which seems to be transported through gap junctions from connected post-mitotic cardiomyocytes [139]. 

The clear demonstration that the post-natal heart retains some proliferative potential has generated new prospects in the field of cardiac regeneration. The ideal regenerative therapy would essentially be able to take advantage of this potential *in situ* and potentiate the progenitor-based renewal that is still present in the adult heart. An alternative approach would be to try to recapture the cardiomyocyte proliferation status of the embryonic/early post-natal period into adult life. 

### 6.2. Persistence of Cardiomyocyte Division after Birth: Is the Key to Regeneration Locked away by microRNAs?

Cardiac regeneration in the zebrafish occurs mainly through the direct division of cardiomyocytes. Unlike mammalian cardiomyocytes, which undergo a final round of division that generates a bi-nucleated cell and from then on cease to perform cytokinesis and tend to become increasingly polyploid, zebrafish cardiomyocytes remain mononucleated and seem to be easily able to re-enter the cell cycle [12]. Interestingly, recent studies have suggested that the mouse heart also retains significant regenerative capacities during the first post-natal week [10]. Porrello and colleagues reported that when the ventricle apex of the heart was amputated in 1 day-old mouse pups (P1), the heart underwent full regeneration without scar formation, similar to adult zebrafish. When similar experiments were performed at P7, the regenerative potential was found to be lost. Cell lineage tracing studies showed that this regeneration is accomplished through cardiomyocyte division, in contrast with the progenitor dependent cell renewal observed in adult mouse hearts [10,13]. A more recent study, however, did not find the same evidence for complete regeneration and reported a reduced level of cardiomyocyte division after injury [140], generating a debate over the origins of the healing ability of the neonatal heart [141,142]. Nevertheless, a recent study in humans has shown that significant rates of cardiomyocyte division are present during the first year of life and can be detectable in young humans up to 20 years old [125]. Therefore, it seems that the mammalian post-natal cardiac switch eliminates the set of more primitive, embryonic characteristics that support regeneration in lower vertebrates. Not surprisingly, considering the prominent role played by miRs in the regulation of this switch, Porrello and colleagues [143] have recently shown that inhibition of the miR-15 family can increase proliferation in the adult mouse heart, leading to improved cardiac function after injury. 

With these observations in mind, Eulalio and colleagues recently performed a high-throughput functional screen to identify miRs able to induce the proliferation of neonatal rat cardiomyocytes using a whole genome miRNA library [144]. The screen identified 204 miRs that strongly increased both DNA synthesis and cytokinesis, of which 40 induced the same effect in mouse neonatal cardiomyocytes, which are known to significantly less proliferative potential. Interestingly, the top 10 miRs promoting cardiomyocyte proliferation in rats were not the same that induced the most efficient proliferation in mouse cells, suggesting that relevant species-specific effects may occur. Two of these miRs (hsa-miR-590 and hsa-miR-199a) were further tested and shown to promote cardiomyocyte proliferation in the heart of adult animals, stimulating marked cardiac regeneration after myocardial infarction with almost complete recovery of cardiac functional parameters. These results suggest that selected miRs can be used as therapeutic tools to revert the cardiomyocyte cell cycle arrest with a positive impact in heart regeneration (Figure 6).

### 6.3. Reprograming of Cardiac Fibroblasts to Functional Cardiomyocytes

A third, more radical hypothesis for *in situ* regeneration is to target cardiac fibroblasts to promote the formation of ventricular cardiomyocytes.

The potential of this idea is underscored by some of the differences in heart structure and response to injury between mammals and other vertebrates with significant cardiac regenerative abilities. In contrast with lower vertebrates, the mammalian heart is part of a high-pressure system that has to deal with significant forces. In agreement with this, the mammalian and lower vertebrate hearts present a significantly different histological organization of the tissue, including a complex network of fibroblasts. Although relatively rare in amphibian and fish, these are the most abundant non-muscle cells in mammals, representing 50% of the cells in the heart. The myocardium of these lower vertebrates is actually much simpler and resembles the embryonic trabecular heart of mammals [145]. 

Upon cardiac injury, the mammalian heart will respond with a strong fibrotic reaction, leading to the quick formation of scar tissue. Bleeding from the heart in a high-pressure circulatory system, which is practically unique to higher vertebrates, can seriously jeopardize survival. Accordingly, some authors have suggested that the limited regeneration potential of the mammalian heart is linked to an evolutionary prioritization of hemostasis and fibrosis [145]. The extensive cardiac fibroblast network of the heart may therefore contribute to create an unfavorable environment for heart regeneration. Strikingly, earlier evidence suggests that even in regenerative animals, fibrosis and regeneration are competing events that need to strike a balance [146]. These observations point to cardiac fibroblasts as important targets in the development of regenerative therapies, either focusing on the control of the fibrotic response, or through the promotion of fibroblast trans-differentiation into cardiomyocytes. This second approach would not only contribute to tip the balance away from fibrotic repair but would also promote regeneration by increasing the rate of cardiomyocyte renewal [147]. 

In agreement with their pervasive functions, miRNAs have also been shown to regulate the fibrotic responses of cardiac fibroblasts. In particular, miR-29, which is down-regulated in response to cardiac injury, has been shown to inhibit the expression of fibrotic genes [148], while miR-21, which is upregulated in response to cardiac stress, has been proposed to promote it [149,150], although a miR-21 KO mouse model raises questions on the essential nature of this response [151].

Finally, although trans-differentiating fibroblasts to cardiomyocytes may sound like a radical approach, the fact is that recent studies have shown it to be possible *in vivo* [152,153]. The possibility of reprograming fibroblasts into muscle cells has been demonstrated over 25 years ago by the forced expression of the muscle transcriptional regulator MyoD [154]. Similarly, these recent studies used retroviral vectors to induce the expression of cardiogenic transcription factors in cardiac fibroblasts *in vivo*, resulting in the differentiation of these cells into mechanically coupled cardiomyocytes. Interestingly, cardiac regeneration in zebrafish has recently been shown to involve a ‘natural’ reprogramming event whereby atrial cardiomyocytes trans-differentiate into ventricular cells [155]. It is worth noting that these studies were performed in zebrafish embryos and therefore it remains to be seen if such a phenomenon could be of significance in another context. Although the potentiation of similar processes in the mammalian heart may therefore not be such a far-fetched approach to promote sustained regeneration after injury, it is also worth noting that the consequences of depleting some of the adult heart population of fibroblasts or atrial cardiomyocytes in favor of ventricular cardiomyocytes are not clear.

Although it has been argued that miRNAs act mostly as a secondary fail safe mechanism in the definition of cell fate, conferring accuracy and robustness to the underlying gene expression programs [46], recent studies have highlighted the tremendous power of these molecules to promote differentiation into specific cell types, including reprogramming of fibroblast into cardiomyocytes [15,90]. 

The observation that defined sets of transcription factors could be used to reprogram fibroblasts to pluripotent stem cells, which could then be differentiated into the cell type of interest, opened the conceptual possibility for direct somatic reprogramming to a desired cell type. The conversion of fibroblasts to cardiomyocytes without an intermediate de-differentiation step was first reported *in vitro* by the Srivastava group [156]. This work was later followed by two studies that demonstrated the feasibility of the approach *in vivo* and reported the functional integration of the newly formed cardiomyocytes into the heart with positive effects in the recovery of myocardial infarction [152,153]. The demonstration that sets of miRs can induce reprogramming of somatic cells to pluripotency [86] established a similar parallel that still has to be systematically explored. The first report of an equivalent reprogramming event used an ‘educated guess’ approach to test the individual and combined effects of six miRs with reported cardiac functions (miR-1; miR-126-3p; miR-133a; miR-138, miR-206; miR-208a) to induce fibroblast trans-differentiation [90]. This led to the identification of an optimal combination of three miRs (miRs-1, 133, 208), together with miR-499, to induce efficient trans-differentiation of cardiac mouse fibroblasts both *in vitro* and *in vivo* (Figure 6). Furthermore, in order to confirm that this effect did not come from the activation of cardiac progenitor cells, the ability of this set of miRs to induce cardiomyocyte formation was tested on mouse tail fibroblasts, confirming the nature of the postulated cell conversion.

## 7. Conclusion and Future Perspectives

The past few years have generated revolutionary insights not only into our understanding of the genetic regulatory programs that control cell function and fate in the context of heart development, but also in our ability to manipulate these programs for therapeutic purposes. Central to these developments is the identification of a previously hidden, non-coding layer for gene expression regulation, of which miRs represent a critical part. Standing together with novel major advances regarding our understanding of stem and progenitor cell function and regulation and of cellular reprogramming events, these progresses herald a new era for the development of regenerative therapies, with particular focus on the heart. Our current understanding suggests that the mammalian heart contains an untapped potential for regeneration that could be engaged to promote new therapies for cardiac injury. Coupled to their physical characteristics, miRs stand as prime candidates for the development of effective tools to promote such *in situ* regeneration. miRNA mimics or inhibitors can be easily synthesized and in animal are easily administered to cells via lipid-based transfection with low toxicity models. Moreover, the small size of a single miRNA allows the easy packing of distinct molecules as required to induce the desired cell response. Although there is still a long way ahead, the recent advances can easily make us believe in a not so far away future, where such therapies will become available for patients. This will however require a deeper understanding of the precise functions played by specific miRNAs in cardiac cell differentiation that will not only require the profiling of miR expression under various developmental, functional, mutant and disease conditions, but also systematic studies focused on target identification based on miRNA-mRNA interactions. The recent developments in deep-sequencing, namely in single-cell sequencing, will provide an important technological basis for such studies, in particular by supporting a much needed characterization of cell type specific expression and function of miRNAs.
